# Supplementary material for: Set4 regulates stress response genes and coordinates histone deacetylases within yeast subtelomeres
Source: Life Sci Alliance. 2021 Oct 8;4(12):e202101126. doi: 10.26508/lsa.202101126 (PMC8507492; doi:10.26508/lsa.202101126)
Supplement: Supplementary file 3 [file LSA-2021-01126_TableS3.docx]

Table S3. Yeast strains used in this study

| Strain | Background | Genotype | Source |
| --- | --- | --- | --- |
| yEG001 | BY4741 | *MATa his3Δ1 leu2Δ0 met15Δ0 ura3Δ0* | YKO |
| yEG322 | BY4741 | *MATa set4Δ::HIS3MX* | (Tran *et al.*, 2018) |
| yEG513 | BY4741 | *MATa set4::FLAG-SET4* | (Tran *et al.*, 2018) |
| yEG873 | BY4741 | *MATa SIR3-HA::NATMX* | This study |
| yEG874 | BY4741 | *MATa set4Δ::HIS3MX SIR3-HA::NATMX* | This study |
| yEG956 | BY4741 | *MATa RPD3-FLAG::KANMX* | This study |
| yEG1010 | BY4741 | *MATa set4Δ::HIS3MX RPD3-FLAG::KANMX* | This study |
| yEG917 | BY4742 | *MATα sir2Δ::KANMX* | This study |
| yEG997 | BY4741 | *MATa set4Δ::HIS3MX sir2Δ::NAT3MX* | This study |
| yEG919 | BY4741 | *MATa his3Δ1 leu2Δ0 met15Δ0 ura3Δ0* | This study |
| yEG920 | BY4741 | *MATa set4Δ::HIS3MX* | This study |
| yEG921 | BY4742 | *MATα rpd3Δ::KANMX* | This study |
| yEG922 | BY4742 | *MATα rpd3Δ::KANMX set4Δ::HISMX* | This study |
| yEG1156 | BY4742 | *MATα his3Δ1 leu2Δ0 met15Δ0 ura3Δ0* | This study |
| yEG1157 | BY4741 | *MATa upc2Δ::KANMX set4Δ::HISMX* | This study |
| yEG1158 | BY4742 | *MATα set4Δ::HISMX* | This study |
| yEG1159 | BY4741 | *MATa upc2Δ::KANMX* | This study |
| yEG017 | W303 | *MATa leu2-3,112 ura3-1 his3-11,15 trp1-1 ade2-1 can1-100 URA3-VIIL* | Paul Kaufman |
| yEG910 | W303 | *MATa set4Δ::KANMX URA3-VIIL* | This study |
| yEG909 | W303 | *MATa set3Δ::KANMX URA3-VIIL* | This study |
| yEG392 | W303 | *MATa leu2-3,112 ura3-1 his3-11,15 trp1-1 ade2-1 can1-100 URA3-VIIL set1Δ::HIS3MX* | This study |
| yEG091 | BY4741 | *MATa set2Δ::HISMX* | This study |
