# Supplementary material for: Set4 regulates stress response genes and coordinates histone deacetylases within yeast subtelomeres
Source: Life Sci Alliance. 2021 Oct 8;4(12):e202101126. doi: 10.26508/lsa.202101126 (PMC8507492; doi:10.26508/lsa.202101126)
Supplement: Supplementary file 4 [file LSA-2021-01126_TableS4.docx]

Table S4. Oligos used in this study

| Gene | Analysis | Sequence |
| --- | --- | --- |
| *COS12* | RT-qPCR | 5’-CATTACAAATACTCCGGGTATAGACA-3’  5’-GCAGCTGGAACCATCAAAA-3’ |
| *YGL262W* | RT-qPCR | 5’-GAGAATTACTCTGACATTGGAGATGA-3’  5’-TTGTCATTACAGAAGCCATCAAC-3’ |
| *YPS5* | RT-qPCR | 5’-CCTCCACAAACGGTGTACCT-3’  5’-TTGCAATAGGCAATGTCAGC-3’ |
| *PAU11 ORF* | RT-qPCR,  chIP | 5’-CGCAACCACCACTCTAGCTC-3’  5’-TGAGCCAAGTGAGCTCTGAT-3’ |
| *PAU13 ORF* | RT-qPCR,  chIP | 5’-TGAAACCTACCCGGTTGAAG-3’  5’-GGGGCAATACCAGTCAACAT-3’ |
| *PAU21/22* ORF* | RT-qPCR,  chIP | 5’-CTTGGTCGAATTGGGTGTTT-3’  5’-TCTGTTGGATGAGCTGCTTG-3’ |
| *ERG11* | RT-qPCR | 5’-CCTCTTATTCCGTCGGTGAA-3’  5’-TGTGTCTACCACCACCGAAA-3’ |
| *TEL07L* | chIP | 5’-AGCCCGAGCCTGTACTAAAT-3’  5’-CAAAAGAAACTTTTCATGGCA-3’ |
| *TEL07L boundary* | chIP | 5’-AGCCATGCGGAAGTTATTTT-3’  5’-TCGACAATAAATAACGCATCG-3’ |
| *PAU11 promoter* | chIP | 5’-CGGGTATAAATAGAGCTGCTTCA-3’  5’-TGCTGGTATAAGCTTAACAGGAAAG-3’ |
| *PAU13 promoter* | chIP | 5’-GATGACTGATGAAGGCATGG-3’  5’-GCTTAACAGGAAGGGAAGGAA-3’ |
| *PAU21/22* promoter* | chIP | 5’-GTGATCATGAAGTTGTGGGAAA-3’  5’-CGATTCGTTAACAGATGCTCCT-3’ |
| *CTT1 promoter* | chIP | 5’-ATTCGACGTAGCCTGGACAC-3’  5’-TGGAATAGAGGTAAAGCAACGA-3’ |
| *PNC1 promoter* | chIP | 5’-TTCAAGGGGCAGGGGTTT-3’  5’-TATTAGCACATCATAATCGTATCTGGA-3’ |
| *ERG3 promoter* | chIP | 5’-CCGATGGCTGCGATAAACGA-3’  5’-TCGCTGCTGAACCTCTTGTT-3’ |
| *ERG11 promoter* | chIP | 5’-TTGCCGGGTTGGACAATCTT-3’  5’-TCGTTTCGTTTAGGGCCAGC-3’ |
| *CENXV* | chIP | 5’-TGCTTTCATAATACCCCACGA-3’  5’-AGGCAAAGGACGCACATATCT-3’ |
| *PRP8 ORF* | chIP | 5’-TTAGAGAAGCCATTGTTGCCA-3’  5’-CCAAGTTTGACACGGTTTTGA-3’ |

**PAU21/22* primers recognize both *PAU21* and *PAU22* due to repetitive sequence.
